# Exploring the Genetic Basis of Adaptation to High Elevations in Reptiles: A Comparative Transcriptome Analysis of Two Toad-Headed Agamas (Genus *Phrynocephalus*)

**DOI:** 10.1371/journal.pone.0112218

**Published:** 2014-11-11

**Authors:** Weizhao Yang, Yin Qi, Jinzhong Fu

**Affiliations:** 1 Chengdu Institute of Biology, Chinese Academy of Sciences, Chengdu, China; 2 University of Chinese Academy of Sciences, Beijing, China; 3 Department of Integrative Biology, University of Guelph, Guelph, Ontario, Canada; State Natural History Museum, Germany

## Abstract

High elevation adaptation offers an excellent study system to understand the genetic basis of adaptive evolution. We acquired transcriptome sequences of two closely related lizards, *Phrynocephalus przewalskii* from low elevations and *P. vlangalii* from high elevations. Within a phylogenetic framework, we compared their genomic data along with green anole, chicken and Chinese softshell turtle, and identified candidate genes and functional categories that are potentially linked to adaptation to high elevation environments. More than 100 million sequence reads were generated for each species via Illumina sequencing. A *de novo* assembly produced 70,919 and 62,118 transcripts for *P. przewalskii* and *P. vlangalii*, respectively. Based on a well-established reptile phylogeny, we detected 143 positively selected genes (PSGs) along the *P. vlangalii* lineage from the 7,012 putative orthologs using a branch-site model. Furthermore, ten GO categories and one KEGG pathway that are over-represented by PSGs were recognized. In addition, 58 GO categories were revealed to have elevated evolutionary rates along the *P. vlangalii* lineage relative to *P. przewalskii*. These functional analyses further filter out PSGs that are most likely involved in the adaptation process to high elevations. Among them, ADAM17, MD, and HSP90B1 likely contributed to response to hypoxia, and POLK likely contributed to DNA repair. Many other candidate genes involved in gene expression and metabolism were also identified. Genome-wide scan for candidate genes may serve as the first step to explore the genetic basis of high elevation adaptation. Detailed comparative study and functional verification are needed to solidify any conclusions. High elevation adaptation requires coordinated changes in multiple genes that involve various physiological and biochemical pathways; we hope that our genetic studies will provide useful directions for future physiological or molecular studies in reptiles as well as other poikilothermic species.

## Introduction

Understanding the genetic basis of adaptive changes is one of the central goals in evolutionary biology [1], and organisms living in extreme environments often provide the best study systems [2]. High elevation environments impose considerable physiological challenges to their residents, particularly these associated with low temperature, low oxygen density, and strong ultraviolet (UV) radiation [3], [4]. In order to survive these extreme stressors, high elevation adaptation may require coordinated structural and transcriptional changes in multiple genes that interact at different levels and involve various physiological and biochemical pathways [3]. Therefore, elucidating how animals cope with high elevation environments provides unique insights in the process of adaptive evolution, especially its intertwined genetic and regulatory basis.

A number of studies have revealed aspects of the genetic mechanisms for high elevation adaptation, particularly for endothermic species [4]. Genes associated with response to hypoxia appear to play a key role in the adaptation process. For example, several studies of Tibetan human populations reported that genes EGLN1, PPARA, and EPAS1, all part of the hypoxia-inducible factor (HIF) pathway, were involved in high elevation adaptation [5], [6], [7]. For other high elevation mammals, Qiu *et al.* [8] screened the genome of yak (*Bos grunniens*), and detected signatures of positive selection for three HIF pathway related genes, ADAM17, ARG2, and MMP3. Ge *et al.* [9] also identified a set of candidate genes in the Tibetan antelope (*Pantholops hodgsonii*) that were under positive selection and likely associated with the HIF pathway, including CCL2 and PKLR. Studies of poikilothermic species are few. Yang *et al*. [10] compared the transcriptomes of the plateau frog (*Rana kukunoris*) and its low elevation relative *R. chensinensis*, and found that genes related to oxygen binding may have been involved in high elevation adaptation, but there was no evidence of involvement among the HIF pathway genes. Poikilotherms represent the majority of animal biodiversity, and they differ from endotherms both physiologically and behaviourally [11]. To generate widely applicable hypotheses, more studies on poikilotherms are needed.

Reptiles are excellent model systems for studying high elevation adaptation of poikilothermic organisms. Similar to mammals, reptiles are terrestrial amniotes and spend most of their time on land. Frequently observed basking behaviour in reptiles also increases their UV exposure [11]. Several toad-headed agamas (genus *Phrynocephalus*) are true high elevation dwellers of the Tibetan Plateau, and live at elevations as high as 5,300 m above sea level (a.s.l.) [12]. Recent phylogenetic studies found that all high elevation species formed a monophyletic group, which nested within the low elevation species [13]. This relationship suggests that the high elevation species may have evolved from low elevation ancestors. Therefore, a comparison between the high elevation species and low elevation species may provide information regarding the high elevation adaptation process. *Phrynocephalus vlangalii* is a high elevation species and primarily distributed in the Tibetan Plateau with altitudes of 2,000–4,600 m a.s.l. It possesses a series of physiological traits that likely represent adaptation to high elevation environments, including remarkably high hemoglobin concentration, hematocrit, mean corpuscular hemoglobin concentration, heart weight to body mass ratio, myocardium capillary density, and succinate dehydrogenase activity [14]. On the other hand, *P. przewalskii* is widely distributed in northern China and Mongolia and mainly occurs at low altitudes of 500–1,500 m a.s.l. [12]. A comparison between them may reveal the genetic basis of high elevation adaptation in reptiles.

The objective of this study is to identify candidate genes and gene functions that may have facilitated adaptation to high elevation environments in *Phrynocephalus* lizards. We sequenced transcriptomes of *P. przewalskii* and *P. vlangalii*, and acquired genomic data of three other reptile species from online databases. Using a branch-site model within a phylogenetic framework, genes that might have experienced positive selection along the *P. vlangalii* lineage were identified. Furthermore, gene functional categories that revealed an accelerated evolutionary rate or were over-represented by positively selected genes were also identified.

## Materials and Methods

### Ethics statement

All animal specimens were collected legally. Animal collection and utility protocols were approved by the Chengdu Institute of Biology Animal Use Ethics Committee.

### Sample collection

Six different tissue types (brain, liver, heart, muscle, and testicle/ootheca) from six individuals (three males and three females) of each species were sampled in order to obtain as many expressed genes as possible. Samples of *P. przewalskii* were collected from the vicinity of Yinchuan City, China (106.87°E, 38.32°N) with an altitude of 1,153 m a.s.l. Samples of *P. vlangalii* were collected from the Zoige County, Sichuan Province, China (102.48°E, 33.72°N) with an altitude of 3,464 m a.s.l. Lizards were captured by hand and euthanized on-site by intracoelomic injection of overdose pentobarbital solution, typically within one hour of capture. Tissue samples were removed and stored in Sample Protector (*Takara*) immediately following euthanasia and dissection.

### Illumina sequencing and *de novo* assembly

RNA was extracted separately from each tissue according to the TRIzol protocol (*Invitrogen*) and then mixed using approximately the same quantity. A single cDNA library was constructed for each species. mRNAs were purified from total RNA by poly (T) oligo-attached magnetic beads (*Life Technologies*). Random oligonucleotides and M-MuLV Reverse Transcriptase (RNase H-) were used to synthesize the first cDNA strand, and the second cDNA strand was synthesized using DNA Polymerase I and RNase H. The cDNA library with an insert size of ∼200 base pairs (bps) was targeted and purified with AMPure XP beads system (*Beckman Coulter*), and subsequently sequenced on an Illumina HiSeq 2000 platform. Paired-end reads were generated with a read length of 100 bps. Both cDNA library construction and Illumina sequencing were carried out by *Novogene* (Beijing, China).

The raw sequence reads were first cleaned by filtering the exact duplicates from both sequencing directions. Subsequently, the sequence reads were trimmed using Trimmomatic [15] by removing adapter sequences, sequences with unknown base call (N) more than 5%, and low quality sequences (<Q20). Reads likely derived from human and *Escherichia coli* contaminants were also filtered using Bowtie [16],[17].

*De novo* assembly of clean reads was performed using a combination of multiple K-mer lengths and coverage cut-off values [18],[19]. We selected five K-mer lengths (21, 31, 41, 51, and 61) and six coverage cut-off values (2, 3, 6, 10, 15, and 20), and generated 30 raw assemblies for each species using ABYSS [20],[21]. A final assembly was produced by eliminating redundancies and integrating sequence overlaps for each transcriptome using CD-HIT-EST [22] and CAP3 [23]. All clean reads were mapped back to the final assembly using Bowtie, and single nucleotide variable (SNP) sites were identified using SAMtools pipeline [24]. For all SNP sites, the base call with the most mapped reads was chosen as the consensus using an in-house Python script.

### Orthology determination and dataset construction

We selected another three closely related vertebrate species to construct our orthologous gene dataset for comparison, including the green anole (*Anolis carolinensis*), the Chinese softshell turtle (*Pelodiscus sinensis*), and chicken (*Gallus gallus*). These three species have well-annotated genomic data, which formed the foundation for orthlology determination and gene function analysis.

We used an analytical pipeline that identifies only single-copy orthologous genes in a relatively conservative fashion. The coding sequences of a 1∶1∶1 orthologous gene dataset shared by *A. carolinensis*, *G. gallus*, and *P. sinensis* was first downloaded from bioMart (Ensembl Genes 74). A best reciprocal hit (BRH) method [25] was then applied to identify 1∶1 orthologs from the final transcriptome assemblies of *P. przewalskii* and *P. vlangalii*. We performed the first tBlastx search using the orthologous coding DNA sequences (CDS) of *A. carolinensis* against each assembly (e-value threshold of 1e-10), and a second tBlastx search with each assembly against the full CDSs of *A. carolinensis*. Only sequences with a significant BRH on the same CDS were considered orthologs [26].

The identified orthologous sequences of *P. przewalskii* and *P. vlangalii* were added to the above 1∶1∶1 orthologous gene dataset and aligned using the “codon alignment” option in Prank [27],[28]. The alignments were further trimmed using Gblocks [29] to remove unreliable regions with “codons” option (“-t = c”) and the default parameters. Sequences with unexpected stop codons and alignment length less than 200 bps were discarded to reduce the chance of false-positive prediction. A saturation test was also performed for all orthologs to remove sequences with saturation at synonymous sites [30]. All third codon position sequences of each orthologous gene were extracted and used to estimate branch lengths of the species tree with the general time reversible model and the program BASEML (in PAML package [31]). If any branch length ≥ 1, the gene was considered saturated, and was discarded from further analysis.

### Candidate gene and gene function identification

**Figure 1:**
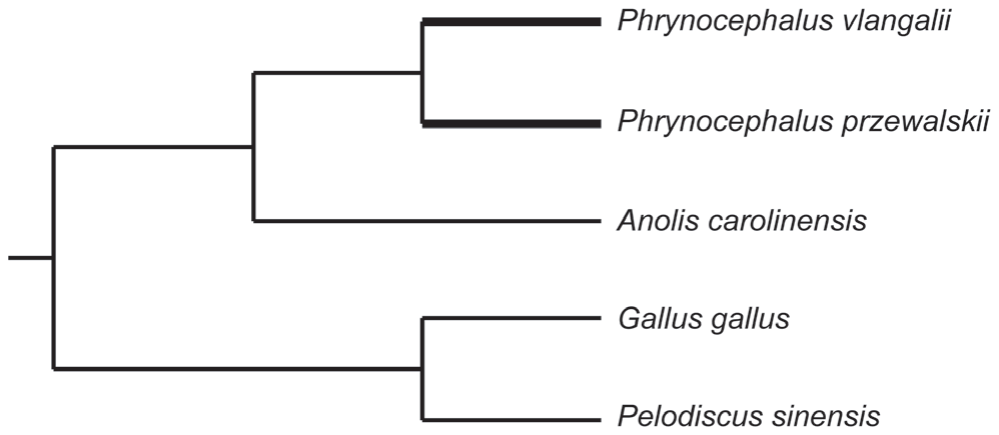
A composite phylogeny of the five vertebrate species examined. The bold lines represent the two *Phrynocephalus* species. The unrooted tree used in CODEML is (((*P. vlangalii, P. przewalskii*), *A. carolinensis*), *G. gallus*, *P. sinensis*).

A composite phylogeny of the five vertebrate species was constructed based on well-established phylogenetic hypotheses (Figure 1) [13],[32]. Based on this phylogeny, we assumed that evolution along the *P. vlangalii* lineage likely represented an adaptation process to a high elevation environment. Genes that have experienced positive selection (or positively selected genes; PSGs) along this lineage were likely involved in high elevation adaptation, and therefore were considered candidate genes. In addition, gene functional categories that have elevated evolutionary rates along this lineage likely have experienced either positive selection or relaxed purifying selection [8],[33]. Gene Ontology (GO) [34] and the Kyoto Encyclopedia of Genes and Genomes (KEGG) [35] pathways were used to define gene functions.

#### Positively selected genes

We used a branch-site model implemented in the program CODEML (in PAML package [31]) to identify PSGs. The *P. vlangalii* lineage was set as the foreground branch, and the optimized branch-site model was used. A likelihood ratio test was conducted to compare the model with positive selection on the foreground branch to a null model with no positive selection on the foreground branch for each orthologous gene [36],[37]. PSGs were inferred only if their *P* values were less than 0.05.

We also identified GO categories and KEGG pathways that were over-represented by PSGs. Functional annotation of PSGs was performed using the DAVID pipeline [38]. Both the enrichment *P*-value and enrichment score (ES) for each category, which is equivalent to the geometric mean of all the enrichment *P*-values of each annotation term that genes in the category are involved in, were calculated. All *P*-values were estimated with a modified Fisher's exact test. Categories with ES greater than 1.3 and *P*-value less than 0.05 were considered over-represented by PSGs [38],[39]. Corrections for multiple tests were also applied in the DAVID pipeline. Similarly, the KEGG pathways that were over-represented by PSGs were also identified using the DAVID pipeline.

#### Elevated evolutionary rate

We used the Ka/Ks ratio to measure the evolutionary rate along a lineage. The values of Ka, Ks, and Ka/Ks ratio were estimated using the *free-ratio* model in CODEML for the *P. przewalskii* branch and *P. vlangalii* branch [35]. The lineage-specific mean values were estimated by concatenated alignments from all orthologs. A comparison of evolutionary rates based on non-synonymous substitution between *P. przewalskii* and *P. vlangalii* was conducted using a binomial test (see [33] for detailed method). Functional categories that had experienced a relatively accelerated evolution were identified. Only GO categories with more than 20 orthologs were included in the analyses, and a Holm test [40] was applied to correct for multiple comparisons.

### Sanger sequencing confirmation

To confirm the accuracy of the Illumina sequencing and assemblies, we randomly selected 20 fragments and re-sequenced them using Sanger sequencing. The lengths of target sequences were limited to below 500 bps to reduce the possibility of sequences spanning across exon boundaries. Primers for PCR amplification and sequencing were designed using Primer3 [41]. Standard PCR with optimized annealing temperature was conducted and the PCR products were directly sequenced. Sequencing was carried out with BigDye chemistry on an ABI 3730 DNA Analyzer. The primer information and PCR conditions are provided in Table S1.

## Results

### Illumina sequencing and *de novo* assembly

**Figure 2:**
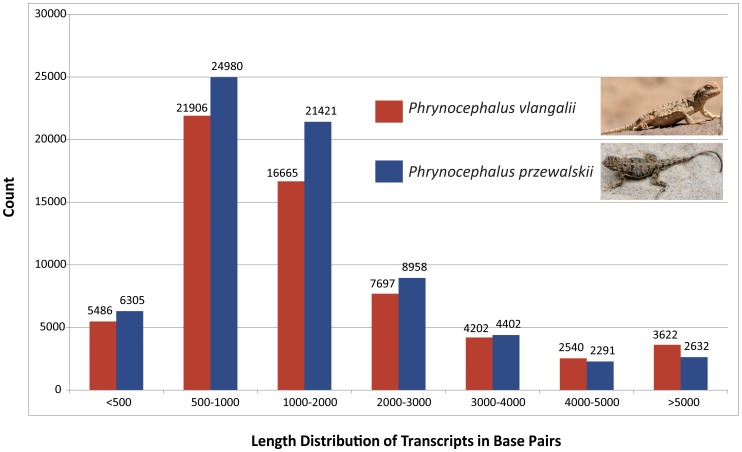
Length distribution of assembled transcripts in base pairs. The numbers of transcripts are shown on top of each bar.

A total of 111,576,922 sequence reads of *P. przewalskii* and 108,689,778 sequence reads of *P. vlangalii* were generated. Defective reads, 3,319,392 and 3,750,648 respectively, were first removed, and the *de novo* assembly of clean reads produced final assemblies with 115.9 mega base pairs (Mb) and 111.6 Mb, respectively. For *P. przewalskii*, 70,989 transcripts were obtained with an N50 length of 2,284 bps and a mean length of 1,632 bps. For *P. vlangalii*, 62,118 transcripts were obtained with an N50 length of 2,728 bps and a mean length of 1,796 bps. Detailed information of the sequence data is summarized in Table 1, and the length distribution of assembled transcripts is shown in Figure 2. All original data are deposited in the NCBI Sequence Read Archive repository (Accession Number: SRR1298770 for *P. przewalskii* and SRR1298771 for *P. vlangalii*).

**Table 1:** Summary of transcriptome data for *Phrynocephalus przewalskii* and *P. vlangalii*.

	*P. przewalskii*	*P. vlangalii*
Total number of raw reads	111,576,922	108,689,778
Total number of clean reads	108,257,530	104,939,130
Total sequences of assembly	70,989	62,118
Total length of assembly (Mb)	115.9	111.6
N50 length of assembly (bp)	2,284	2,728
Mean length of assembly (bp)	1,632	1,796
Median length of assembly (bp)	1,124	1,136

bp = base pair; Mb = mega base pair.

Our Sanger sequencing confirmed the accuracy of the Illumina sequences and our final assemblies. For *P. przewalskii*, nine fragments were successfully sequenced with a total length of 2,799 bps, and we found 12 nucleotide sites that were different. Similarly, 15 fragments were successfully sequenced for *P. vlangalii* with a total length of 4,485 bps, and we found three sites that were different (Table S1). Overall, the consistency was high, 99.57% for *P. przewalskii* and 99.93% for *P. vlangalii*. Some of the discrepancies were likely derived from individual variations rather than errors. Five of the sites with different calls were putative SNP sites; when individuals were pooled for Illumina sequencing, only the genome with a dominant amount of RNA would be selected in the final assemblies. Ten of the 12 sites with different calls in *P. przewalskii* were from a single fragment, suggesting our PCR likely amplified more than one target, possibly paralogous genes. Other sources of differences might include RNA editing, sequencing errors, or assembly errors. The high consistency between Sanger sequencing and our assembly confirmed the effectiveness of our methods and the accuracy of the final assemblies.

### Putative orthologous genes

A total of 7,275 putative orthologs were first identified for both species. After removing the low quality and short sequences, 7,012 orthologs were preceded to downstream analyses. Therefore, our final dataset included five species, *P. przewalskii*, *P. vlangalii*, *A. carolinensis*, *G. gallus*, and *P. sinensis*, and 7,012 genes.

### Functional categories with elevated evolutionary rates

**Figure 3:**
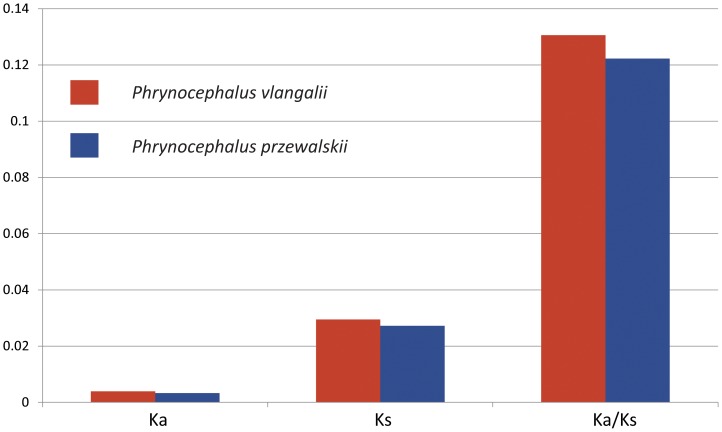
Comparison of evolutionary rates between the *P. vlangalii* and *P. przewalskii* lineages. The mean Ka, Ks, and Ka/Ks ratio of both lineages are presented.

The mean values of Ka, Ks, and Ka/Ks ratio along the *P. vlangalii* lineage were 0.0039, 0.0295, and 0.1306, respectively, and the values along the *P. przewalskii* lineage were 0.0033, 0.0272, and 0.1223, respectively. The *P. vlangalii* lineage demonstrated slightly higher evolutionary rates than *P. przewalskii* (Figure 3).

**Figure 4:**
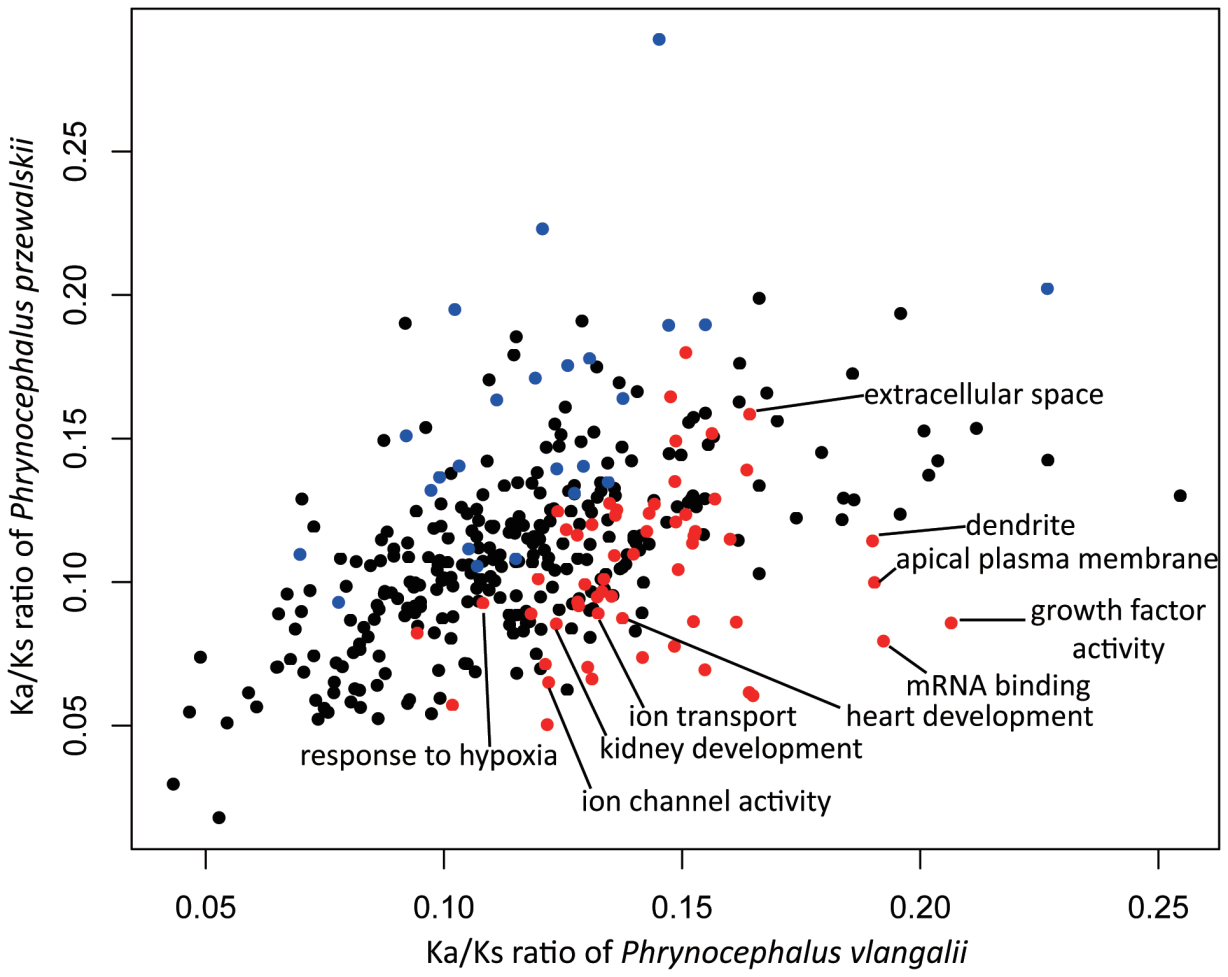
Comparison of Ka/Ks ratios between *P. vlangalii* and *P. przewalskii* by GO functional categories. Blue dots represent categories with an elevated evolutionary rate along the *P. przewalskii* lineage. Red dots represent categories with an elevated evolutionary rate along the *P. vlangalii* lineage. A full list of the categories is presented in Table S2.

A total of 58 GO categories revealed relatively accelerated evolutionary rates (*P*<0.05) in the *P. vlangalii* lineage, compared to *P. przewalskii* (Figure 4 and Table S2). Among them, the majority were involved in ion transport, gene expression, and organ development, such as ion transport (GO: 0006811), ion channel activity (GO: 0005216), negative regulation of gene expression (GO: 0010629), DNA-dependent transcription (GO: 0006351), heart development (GO: 0007507), and kidney development (GO: 0001822). Notably, the category of response to hypoxia (GO: 0001666) revealed an accelerated evolutionary rate in the *P. vlangalii* lineage. Similarly, 24 GO categories revealed relatively accelerated evolutionary rates in the *P. przewalskii* lineage, compared to *P. vlangalii* (Figure 4 and Table S2). Categories involving molecular binding were dominant, such as identical protein binding (GO: 0042802), heme binding (GO: 0020037), and ATP binding (GO: 0005524).

### Positively selected genes

The branch-site model and likelihood ratio test identified 143 PSGs (*P*<0.05) along the *P. vlangalii* lineage (Table S3). Interestingly, three genes from the ‘response to hypoxia’ GO category, MB, ADAM17, and HSP90B1, were among them. MB encodes myoglobin, which is an oxygen-binding hemoprotein, and plays a key role in dealing with chronic hypoxia (e.g. [42],[43]). ADAM17 encodes a crucial regulator of hypoxia-inducible factor-1α (HIF-1α), which is associated with the cellular response to hypoxia [44],[45]. HSP90B1 is one of the heat shock protein members and plays important roles in folding proteins in the secretory pathways [46]. In addition, POLK from the GO category of DNA repair also experienced positive selection. POLK is a well-studied gene, and plays an important role in response to DNA damage [47]. Furthermore, three other PSGs, INPPL1, PPP2CB, and HDAC2, are associated with transcription and gene expression [48],[49],[50].

Based on gene annotation, 10 GO categories were over-represented by 42 PSGs with ESs greater than 1.3 and *P* values less than 0.05, including RNA metabolic process (GO: 0016070, ES = 2.8), gene expression (GO: 0010467, ES = 2.0), ion binding (GO: 0043167, ES = 1.6), and several others (Table 2). These categories were generally in three functional groups: metabolism, ion binding, and gene expression. Similarly, one KEGG pathway (endocytosis, ko04144) was over-represented by PSGs with an ES of 3.3.

**Table 2:** List of GO functional categories that are over-represented by positively selected genes.

ID	GO categories	Count	*P*-value	Enrichment score (ES)
GO: 0016070	RNA metabolic process	7	3.20E-02	2.8
GO: 0010467	gene expression	12	2.20E-02	2.0
GO: 0044267	cellular protein metabolic process	17	2.10E-02	1.7
GO: 0044260	cellular macromolecule metabolic process	26	2.70E-03	1.6
GO: 0046872	metal ion binding	22	1.70E-02	1.6
GO: 0043169	cation binding	22	2.10E-02	1.6
GO: 0043167	ion binding	22	2.20E-02	1.6
GO: 0043170	macromolecule metabolic process	26	1.60E-02	1.5
GO: 0044238	primary metabolic process	30	2.00E-02	1.4
GO: 0044237	cellular metabolic process	28	2.80E-02	1.4

## Discussion

### Transcriptomes of *Phrynocephalus*

We reported transcriptome data for two lizard species. A total of 70,989 transcripts were generated for *P. przewalskii*, and 62,118 transcripts were generated for *P. vlangalii*. Squamates represent one of the most diverse vertebrate groups; however, genomic data of squamates are limited. At present time, only one species (the green anole, *A. carolinensis*) has a completed whole genome sequence [51]. Although the draft genomes of the Burmese python (*Python molurus bivittatus*) and king cobra (*Ophiophagus hannah*) have been recently published, the annotation and orthologous identification is far from being complete [52],[53]. Transcriptome data represent a feasible and inexpensive alternative to whole genome sequencing for many genome level studies. In our case, the two transcriptomes allow us to examine 7,012 orthologous coding genes and their potential involvement in the adaptation process to high elevation environments.

We pooled RNA samples from multiple tissue sources in order to obtain sequences of a large number of expressed genes. With this method, we were able to recover more than 60,000 transcripts with N50 lengths greater than 2,000 bps for each species. Nevertheless, there are certain drawbacks associated with this approach. For example, we were unable to recover any gene expression data, which limited this study to sequence level only. In addition, pooling tissues together may reduce the number of detected transcripts and cause problems in the assembly process, such as in the case of alternative splicing producing different transcripts in different tissues.

### Candidate genes and their functions

The two *Phrynocephalus* species demonstrated relatively low evolutionary rates. Their Ka/Ks ratios are 0.1306 and 0.1223, much lower than those of human (0.208), chimpanzee (0.194), and dolphin (0.237), but similar to mouse (0.142) and rat (0.137) [30],[33]. The low rates suggest that most genes in both lineages were under purified selection. Their large population size may also contribute to their low Ka/Ks ratio [33]. Between the two species, *P. vlangalii* has a slightly higher evolutionary rate than *P. przewalskii*. There are many more GO categories with accelerated evolutionary rates in the *P. vlangalii* lineage (58) than in the *P. przewalskii* lineage (24). This is consistent with our assumption that *P. vlangalii* may have evolved from a low elevation ancestor, and the evolutionary change along the lineage may represent adaptation process to high elevations.

We identified 143 genes that may have experienced positive selection along the evolution of the high elevation *P. vlangalii* lineage. Among them, 42 are in the ten GO categories that are over-represented by PSGs, and 61 are associated with 40 GO categories with accelerated evolutionary rates (Table S3). The over-represented categories are mostly in three functional groups: metabolism, ion binding, and gene expression. The categories with elevated evolutionary rates primarily involve ion transport, gene expression, and organ development. Some gene functions are clearly associated with high elevation adaptation while others are less obvious.

The GO category of response to hypoxia (GO: 0001666) is particularly interesting. Hypobaric hypoxia is one of the main environmental stressors at high elevations, and is generally difficult to mitigate by behavioural avoidance [54]. We found that genes in this category have an elevated average evolutionary rate, suggesting their potential involvement in high elevation adaptation in *Phrynocephalus* lizards. Similar findings were also reported in endothermic species living in high altitude environments, including yak [8] and ground tit [55]. Moreover, three genes in the category, ADAM17, MB, and HSP90B1, have experienced positive selection. ADAM17 encodes a regulator at upstream of the HIF-1α pathway, which affects the stability of HIF-1α by regulating the production of tumor necrosis factor α [44],[45]. HIF-1α is a key regulator of cellular reaction to hypoxia, and it regulates transcription of a wide range of genes associated with energy metabolism, vasodilatation, and angiogenesis [56]. Notably, ADAM17 was also found under positive selection in yak [8], a high-altitude mammal. These results reinforce the inference that this gene plays a role in the adaptation to high elevation environments. MB encodes a hemoglobin-related protein, myoglobin, which is an oxygen-binding hemoprotein and has several key functions in hypoxic conditions, including oxygen transport, oxygen storage, oxygen utilization, and oxygen reduction [42]. Hypoxia-inducible myoglobin is expressed not only in muscle tissues but also in non-muscle tissues, particularly in blood [43]. The MB gene is very likely involved in high altitude adaptation of *P. vlangalii*. Our understanding of hypoxia adaptation of these lizards at phenotypic level is limited, though a recent physiological study of *P. vlangalii* suggested that it has special anatomical, physiological, and biochemical adaptive features to live in a hypoxic environment [14]. No direct link has been established between these features and any of our PSGs.

Strong UV radiation is another major physiological stressor on animals at high altitudes, which may damage DNA molecules by generating highly reactive chemical intermediates such as oxygen radicals [57],[58]. It is particularly acute for reptiles because they often use basking for thermoregulation [11]. One candidate gene, POLK, is in the functional category of DNA repair. It encodes a low-fidelity DNA polymerase, which enables DNA to be synthesized across damaged bases [47]. POLK also involves several other functional categories that are over-represented by PSGs (Table S3). The potential involvement of POLK in high elevation adaptation provides clues of how *P. vlangalii* may resist increased DNA damage.

The adaptation to high altitudes may occur at the transcription and expression level in addition to nucleotide substitutions at the DNA sequence level. Several GO categories involving gene expression in *P. vlangalii* lineage have accelerated evolutionary rates (Table S2). The GO category of gene expression (GO: 0010467) is over-represented by PSGs (Table 2). In addition, three key regulators of transcription and expression, INPPL1, PPP2CB, and HDAC2, are among the PSGs in the *P. vlangalii* lineage. INPPL1 encodes protein that regulates insulin, epidermal growth, and actin [48]. PPP2CB is implicated in a series of biological processes of controlling cell growth and division [49]. HDAC2 encodes an enzyme that is responsible for the deacetylation on the N-terminal of lysine residues of the core histones [50]. These results demonstrate the complexity of the adaptive process to high elevation environments. We did not directly examine gene expression levels due to the limits of our sampling strategy.

### Future directions

Genome-wide scan for PSGs may serve as the first step to explore the genetic basis of high elevation adaptation. It has several advantages over the candidate gene approach. Multiple genes at multiple levels (genes, pathways, functional groups) are examined simultaneously. This allows the interactions of genes to be inferred, which is essential for high elevation adaptation [4]. In addition, without pre-defined targets, it is likely to yield previously unknown pathways or functions. Nevertheless, PSGs do not necessarily represent high elevation adaption. There are many reasons that a gene may experience positive selection. For example, genes associated with the host-pathogen interaction or immune system are frequently under positive selection [59], and they may not specifically contribute to high elevation adaptation. Furthermore, without corroboration from phenotypic data and functional verification, these PSGs are no more than “candidate” genes in any adaptive processes.

For future studies, other reptile groups that live at high-altitude environments should be examined and potential convergent evolution can be investigated. As well, population level comparisons of candidate genes would detect positive selection at a more recent temporal scale. Finally, research at gene expression level and, perhaps more importantly, at physiological (phenotypic) level are essential to understand the adaptation process to high elevation environments.

## Conclusions

Using transcriptome data and a branch-site model, we evaluated positive selection and evolutionary rates of 7,012 genes along the high elevation *P. vlangalii* lineage. A total of 143 candidate genes were identified and several gene functional categories that are likely associated with high elevation adaption were also detected. These functional analyses further sift out candidate genes that are of particular interests. Among them, ADAM17, MD, and HSP90B1 likely contribute to response to hypoxia, and POLK likely contributes to DNA repair. These genes and their function should be the priority for future studies of high elevation adaptation in poikilothermic animals.

High elevation adaptation is a very exciting area of research. Direct examinations of phenotypic traits and a better understanding of their molecular and cellular processes are essential. We hope that our genetic studies will provide useful directions for future physiological or molecular studies in reptiles as well as other poikilothermic species.

## Supporting Information

Table S1 **Information on primers and randomly selected fragments for PCR and Sanger sequencing.**
(XLSX)

Table S2 **List of GO categories with relatively elevated evolutionary rates in the** ***P. vlangalii*** **and** ***P. przewalskii*** **lineages.**
(XLSX)

Table S3 **List of positively selected genes along the high elevation** ***P. vlangalii*** **lineage.**
(XLSX)
